# Closing yield gaps in smallholder goat production systems in Ethiopia and India

**DOI:** 10.1016/j.livsci.2018.06.015

**Published:** 2018-08

**Authors:** Dianne Mayberry, Andrew Ash, Di Prestwidge, Mario Herrero

**Affiliations:** CSIRO Agriculture and Food, Queensland Bioscience Precinct, 306 Carmody Rd, St Lucia, Qld 4069, Australia

**Keywords:** Health, Disease, Nutrition, Grazing, Breeding, Genetic improvement

## Abstract

Small ruminants such as goats are an important source of income for smallholder farmers in South Asia and Sub Saharan Africa: they may be kept as a stepping stone to owning larger and higher-value animals such as cattle or buffalo, or provide a more-profitable and less-risky alternative in marginal or densely populated areas where access to feed resources are limited. However, smallholder goat production in these areas is often low due to low growth and reproduction rates and high animal mortality. The aim of this study was to investigate the potential for different intervention packages to increase yields and profitability of goat meat production in Ethiopia and India. Packages were based on improved nutrition, reduced flock mortality from improved control of health and diseases, and replacing indigenous livestock with improved goat breeds. Household modelling was used to simulate the effects of interventions on goat production and household income in the extensive lowland grazing zone and highland mixed crop-livestock zones of Ethiopia, and the extensive arid zone of India. Our analysis showed that there are opportunities to increase goat meat production in both countries. Reproduction, liveweight gain and survival rates can be increased through better nutrition, genetics and healthcare, but the biggest increase in production and profits occurred when multiple interventions were combined. Importantly, interventions resulting in the biggest increases in goat meat production or number of animals sold did not always give the highest profits.

## Highlights

• Household modelling highlights ways to increase goat production.
• Large increases in goat meat production are possible in Ethiopia and India.
• Increasing goat production did not always increase household income.
• Yield gaps are best addressed by integrated technologies using a systems approach.

## 1 Introduction

Small ruminants (sheep and goats) play an essential role in improving the livelihoods of smallholder farmers in developing countries, providing meat, fibre, milk, skin/leather, manure and short-term cash income. The global population of small ruminants is concentrated in South Asia and Sub Saharan Africa, which are the focus of this paper. We use examples from India and Ethiopia, and focus on goats as a representative model of small ruminants, notwithstanding that sheep are also important in smallholder farming systems. There are around 200 million small ruminants in India (MoA 2014a) and 56 million in Ethiopia (Central Statistics Agency 2015), the vast majority of which (> 98%) are indigenous breeds. In both countries, goats are predominantly kept for meat production and managed in low-input, extensive grazing systems based on communal lands and native pastures (Tesfahun et al., 2017). However, as grazing resources become increasingly scarce, it is becoming more common for farmers to tether or pen their animals (Peacock 2005).

Compared to larger livestock such as cattle and buffalo, small ruminants have many advantages. They require a smaller up-front investment, and their short breeding cycle and fattening times provide a quicker return on investment, can assist with short-term cash flows, and help flocks to recover quickly following drought (Hirpa, 2008, Peacock, 2005). Goats are also more suitable for marginal lands because they require less feed than larger animals, can browse trees and shrubs, and are better able to digest roughages (Desiere et al., 2015). While small ruminants have traditionally been considered a stepping stone to owning higher value animals such as cattle or buffalo, there is evidence that some farmers prefer small ruminants to cattle, especially in densely populated areas with declining feed resources (Desiere et al., 2015). In these areas, keeping a larger number of sheep or goats may be considered less risky for smallholders than owning a small number of valuable cattle.

Despite the advantages of small ruminants, goat producers face many challenges that affect the productivity of their livestock enterprise. The main problems are low productivity and high mortality (especially of offspring). Annual meat production is low, and is often less than 10 kg per animal (Peacock and Sherman, 2010, Sebside, 2008, Vijay and Singh, 2015). This is primarily caused by inadequate nutrition, which results in low growth rates and small mature size, and is compounded by slaughtering of animals at immature body weights (Hegde and Deo 2015). Poor nutrition also contributes to high mortality rates, which are also caused by disease outbreaks. Average annual mortality rates are high at around 10–20% (Singh et al., 2009), but can increase to over 50% during poor seasons and disease epidemics. Consequently, shortage of feed and health issues are often ranked as the most significant constraints to production (e.g., Gizaw et al., 2010, Assen and Aklilu, 2012, Vijay et al., 2014, Suresh and Chaudhary, 2015). Production may also be limited by the genetic potential of unimproved local breeds.

Improvement strategies to lift productivity of goat systems have been developed and include improved animal feeding based on higher quality forages and more efficient utilisation of existing feed resources, control of diseases that affect animal production and survival, and introducing improved meat breeds to cross with low producing indigenous breeds (Gizaw et al., 2010, Hegde and Deo, 2015, Suresh and Chaudhary, 2015). However, there is little information available in the literature about the scale of potential increases in goat production, and the impacts on household income. The aim of this study was to investigate the potential for different intervention packages to increase yields and profitability of goat meat production in Ethiopia and India. This information will contribute to making informed investment decisions and target technologies in the livestock sectors of developing countries (Herrero et al., 2015).

## 2 Methods

### 2.1 Experimental design

Household modelling was used to evaluate strategies to increase goat production within the constraints of the current production systems, and indicate likely economic outcomes. Interventions evaluated in this study included 1) improving goat nutrition, 2) reducing flock mortality through improved control of health and diseases, and 3) replacing indigenous livestock with improved goat breeds.

Baseline scenarios and interventions to increase production were simulated using a smallholder household model run over a 20 year period. The integrated analysis tool (IAT), version 1.3.7 (Lisson et al., 2010) is a spreadsheet model that integrates crop production, forages, livestock production, flock dynamics, household economics and labour supply. It has previously been used to model both intensive (cut and carry) and extensive (grazing) livestock production systems in East Asia (China, Indonesia, Vietnam), South and West Asia (India, Pakistan), and Africa (Burkina Faso, Cameroon, Ethiopia, Niger, Senegal, Zimbabwe) (Komarek et al., 2012, Mayberry et al., 2017, Parsons et al., 2011, Rigolot et al., 2017, Shafiullah, 2012).

### 2.2 Model setup and baseline scenarios

The IAT was parameterised to create baseline scenarios for goat meat production across Ethiopia and India. Household-level data on goat production was obtained from a number of sources. The IMPACT Lite (CGIAR research program on Climate Change Agriculture and Food Security, Rufino et al., (2013)), Living Standards Measurement Study (LSMS, The World Bank) and Village Dynamics in South Asia (VDSA, ICRISAT) datasets and government census data (Central Statistics Agency 2015, Ministry of Agriculture 2014a, Ministry of Agriculture 2014b, Ministry of Agriculture 2015) provided information on the number of animals per household, reproduction, mortality, feeding and production of crops used for livestock feed. Additional data on animal management, production and pricing was gained from the literature (Assen and Aklilu, 2012, Chandran et al., 2013, Chaturvedi et al., 2008, Dereje et al., 2015, Gizaw et al., 2010, Gupta et al., 2014, Hirpa, 2008, Kumar and Kumar, 2013, Nayak et al., 2008, Singh et al., 2009, Tanwar and Chand, 2011, Yadav and Tailor, 2010). Together these reports were able to provide a picture of reproductive rates, mortality rates, weights and ages of sale animals, etc. that permitted model parameterisation.

Consequently, baseline scenarios were developed to reflect characteristics of a typical goat meat production enterprise in each region. Specific details of each baseline scenario are described in Table 1. In Ethiopia, goat meat production was simulated for three agro-ecological zones as defined by the Ethiopia Livestock Master Plan (Shapiro et al., 2015). These were: Lowland Grazing (LG) in pastoral zones (< 900 mm rainfall) based largely on grazing of natural pastures, highland Mixed crop-livestock Rainfall Deficient (MRD) zone where rainfall is 900–1400 mm and households are rural with some crop land and access to grazing, and the highland Mixed crop-livestock Rainfall Sufficient (MRS) zone where rainfall is greater more than 1400 mm. For this study the household type in the MRS was based on peri‑urban and urban livestock producers who do not have access to land. In India, goat production was simulated in the arid zone, which is characterised by erratic rainfall and long dry spells, with a large, but increasingly degraded, grazing resource that supports a large number of small ruminants.

**Table 1:** Characteristics of baseline goat meat production households in different agro-ecological regions of Ethiopia and India, and details of simulated interventions. Feed weights are fresh weights. LG: lowland grazing pastoral zone; MRD: mixed crop-livestock rainfall deficient zone; MRS: mixed crop-livestock rainfall sufficient zone. 1 USD = 22 ETB or 66 INR.

Scenario & interventions	Description	Livestock breed	Number breeders
**Ethiopia – LG**	Lowland grazing zone. Extensive grazing system on 10 ha communal land with native pastures and browse. No supplementation. Baseline flock mortality 25%. Healthcare 9 ETB/head/month. Male offspring sold at 12 months and females kept as replacements.	Local goats	9–18
Improved genetics	Local goats replaced with crossbred goats. Healthcare 11 ETB/head/month.	Crossbred goats	9–25
Low mortality	Flock mortality reduced to 10%. Healthcare 12 ETB/head/month.	Local goats	9–18
Improved genetics + improved pasture	Local goats replaced with crossbred goats. N content of native pasture increased.	Crossbred goats	9–30
Improved pasture	N content of native pasture increased.	Local goats	9–25
Improved genetics + low mortality	Local goats replaced with crossbred goats. Flock mortality reduced to 10%. Healthcare 16 ETB/head/month.	Crossbred goats	9–25
Improved pasture + low mortality	N content of native pasture increased. Flock mortality reduced to 10%. Healthcare 12 ETB/head/month.	Local goats	9–25
Improved genetics, improved pasture + low mortality	Local goats replaced with crossbred goats. N content of native pasture increased. Flock mortality reduced to 10%. Healthcare 16 ETB/head/month.	Crossbred goats	9–30
**Ethiopia – MRD**	Highland mixed farming in rainfall deficient zone. 0.8 ha natural pastures available for grazing. Goats supplemented with cereal straw. Baseline flock mortality 20%. Healthcare 6 ETB/head/month. Male offspring sold at 12 months and females kept as replacements.	Local goats	6–14
Improved genetics	Local goats replaced with crossbred goats. Healthcare 8 ETB/head/month.	Crossbred goats	10–20
Low mortality	Flock mortality reduced to 10%. Healthcare 9 ETB/head/month.	Local goats	6–12
Improved genetics + low mortality	Local goats replaced with crossbred goats. Flock mortality reduced to 10%. Healthcare 11 ETB/head/month.	Crossbred goats	6–12
Improved pasture + low mortality	N content of native pasture increased. Flock mortality reduced to 10%. Healthcare 9 ETB/head/month.	Local goats	6–14
Improved genetics, improved pasture + low mortality	Local goats replaced with crossbred goats. N content of native pasture increased. Flock mortality reduced to 10%. Healthcare 11 ETB/head/month.	Crossbred goats	6–12
**Ethiopia – MRS**	Highland mixed farming in rainfall sufficient zone. No cropping land. Goats fed cereal straw and legume hay. Baseline flock mortality 15%. Male offspring sold at 12 months and females kept as replacements. Healthcare 8 ETM/head/month.	Crossbred goats	6–10
Low mortality	Flock mortality reduced to 7.5%. Healthcare 11 ETB/head/month.	Crossbred goats	6–10
Improved forage + low mortality	Flock mortality reduced to 7.5%. Healthcare 11 ETB/head/month. Males supplemented with noug cake at 0.2 kg/head/day.	Crossbred goats	6–10
**India – arid zone**	Arid zone. Restricted grazing of native pastures with no supplementation. Baseline flock mortality 20%. Healthcare 10 INR/head/month. Male offspring sold at 6 months and females kept as replacements.	Local goats	8–10
Low mortality	Flock mortality reduced to 10%. Healthcare 20 INR/head/month.	Local goats	8–10
Free grazing	Flock size reduced by half to allow unrestricted access to feed.	Local goats	4–6
Supplement kids	Kids supplemented with wheat bran at 0.2 kg/head/day and sold at 10 months.	Local goats	8–10
Supplement does (straw)	Does supplemented with cereal straw at 0.5 kg/head/day.	Local goats	8–10
Improved pasture	N content of pasture increased (to simulate reseeding with legumes).	Local goats	8–10
Improved pasture + low mortality	N content of pasture increased. Mortality reduced to 10%. Healthcare 20 INR/head/month.	Local goats	8–10
Supplement does (bran)	Does supplemented with wheat bran at 0.2 kg/head/day.	Local goats	8–10

The livestock simulation model within the IAT predicts the liveweight gain and reproduction cycles for ruminant livestock under specified local feeding and husbandry practices. Livestock production is based on energy and protein supply in the diet using the Feeding Standards for Australian Livestock (Freer et al., 2007). Default livestock breeds are available within the model, and we edited these to reflect the mature size and characteristics of local breeds; local goats (30 kg) and crossbred goats (35 kg) in Ethiopia, and local goats (25 kg) in India. Flock size is based on a minimum and maximum number of females of breeding age (breeders) set by the user, with management rules to sell livestock based on age and weight of different livestock classes.

For the modelling we assumed that goats are predominantly fed through grazing of pastures, but in more intensive systems may be tethered or penned and fed forages, crop resides, crop by-products and purchased supplements. Native pastures and communal lands provided the main source of feed for goats in the grazing scenarios. Grazed pastures were simulated using GRASP (McKeon et al., 1990), driven by daily climate variables simulated for current climate within the study regions using the MarkSim weather generator (Jones and Thornton, 2000). There was insufficient climate and soil information available to model crop production, so yields and monthly availability of crops (grain and residues) and improved forages were estimated based on information available in the databases and papers described above. Crop residues were stockpiled at harvest and available until the supply was exhausted, when additional feeds were purchased if necessary.

We specifically included profit from goat production in our analysis because profit and financial risk are key considerations for farmers. Annual profit (income minus expenses) is calculated by the IAT model, and was only considered for the livestock component of the farming system. We did not include crop incomes and expenses. Income was gained from the sale of offspring and culled breeders (Table 2). Costs included health care and purchasing feed for livestock if feed production on-farm was insufficient. It was assumed there were no costs in producing livestock feed on-farm because the majority of feeds are either by-products of crop production (straw, stovers, brans) or cut/grazed from communal lands.

**Table 2:** Livestock management costs (excluding feed) and sale prices for live animals. LG: lowland grazing pastoral zone; MRD: mixed crop-livestock rainfall deficient zone; MRS: mixed crop-livestock rainfall sufficient zone. 1 USD = 22 ETB or 66 INR.

Country x Breed	Costs	Income from sale of goats	
	Healthcare Per head per month	Female Per kg liveweight	Male Per kg liveweight
*Ethiopia (ETB)*			
Local goats-LG	9	20–30	35
Local goats-MRD, MRS	6	20–40	40
Crossbred goats-LG	11	20–30	35
Crossbred goats-MRD, MRS	8	20–40	40
*India (INR)*			
Local goats	10	130	160

The costs of labour from hiring outside of the family was not explicitly considered in our analyses because there was insufficient information available in the literature to parameterise the model. It was assumed that enough family labour was available to sustain livestock production.

### 2.3 Interventions to increase goat meat production

#### 2.3.1 Improved nutrition

We investigated several options for improving livestock nutrition. For extensive goat production we explored increasing both the quality and quantity of feed resources. To improve the quality of communal grazing lands we simulated reseeding of natural pasture with a perennial, herbaceous legume (e.g., Stylosanthes) by increasing the N content of the available forage by 0.5% N. The seasonal decline in nitrogen content of pasture was also reduced to simulate the higher protein content maintained in grass-legume pastures when grasses mature and senesce. It is recognised that augmentation of native pastures with a legume will not be relevant to all systems, but it can be a relatively low cost way of improving the feedbase. The costs of establishing an improved pasture are usually borne by the farmer, but because pasture areas are communal grazing lands it was assumed that the government would provide the investment for pasture improvement and no cost to the producer was included in our modelling. In the baseline goat production scenarios we restricted the amount of feed goats were able to consume to mimic degradation of rangelands and competition for feed resources. In the India free grazing scenario we investigated increasing feed available for grazing by decreasing the number of animals livestock owned by a farmer as a proxy for reduced overall stocking rates (Table 1).

In more intensive production scenarios, nutrition was primarily improved by increasing the amount and quality of supplements offered to different classes of livestock (kids, does). Supplements included crop residues and crop by-products (e.g., noug cake and wheat bran). In cases where enough feed could not be grown on-farm, additional feeds were purchased. Quality and costs of supplements are described in Table 3. Feed was not offered ad libitum as our experience is that this is not common in smallholder farming systems.

**Table 3:** Feed quality parameters used by the IAT model and prices of purchased feed (fresh weight basis). 1 USD = 22 ETB or 66 INR.

Feed type	Dry matter (%)	Dry matter digestibility (%)	N content (% DM)	Cost per kg
*Ethiopia*				*ETB*
Cereal straw	90	45	0.7	0.3
Noug seed cake	90	70	5.1	1.8
Urea-treated stover	90	57	3	0.6
Pulse straw	90	55	1.8	0.5
*India*				*INR*
Cereal straw	90	46	0.7	5
Wheat bran	90	69	2.8	16

#### 2.3.2 Reduced mortality

While the range of diseases that affect goats has been well documented (e.g., Gizaw et al., 2010) and mortality rates can be high at 20–30%, there is a lack of published data on mortality rates of specific pests or diseases. There is even less information published on the production losses (reduced growth and/or reproduction) from various pests and diseases. Consequently, in this study a generic approach was adopted whereby the baseline level of animal livestock mortality was decreased and vet/healthcare costs were increased based on information available in the literature (Perry et al., 2001), assuming a complex of diseases. Cost of healthcare is described in Table 2.

#### 2.3.3 Improved genetics

Replacing local livestock breeds with crossbreds was only investigated for the Ethiopia production systems. Improved breeds have higher production potential and sale value, but also higher liveweight, feed requirements and production costs (Kumar and Kumar, 2013, Leroy et al., 2016; MoA 2014b). The IAT model was parameterised for crossbreds assuming a higher mature body weight than local breeds.

## 3 Results

### 3.1 Ethiopia

In the lowland region, improving forage quality through reseeding communal grazing land with a legume dramatically increased productivity and profit for both local breeds and crossbred goats. This was achieved through being able to carry more livestock and producing more liveweight per head. A combination of crossbred goats, legume addition and improved livestock health (reduced mortality) resulted in the highest production and profits.

In the MRD zone, introducing crossbred livestock alone was not sufficient to improve profitability because productivity was still constrained by high mortalities (25%) and low reproduction rates. Even when the improved healthcare intervention was included, mortality rates were still high at 17%. However, improving nutrition through better forages resulted in reduced mortality rates, a three-fold increase in productivity, and a shift from financial losses to significant profit (Table 4).

**Table 4:** Average annual productivity and profit for baseline scenarios and modelled interventions to increase goat meat production in Ethiopia and India. Scenarios are ranked by production (liveweight of goats available for sale or consumption by household) within each site. LG: lowland grazing pastoral zone; MRD: mixed crop-livestock rainfall deficient zone; MRS: mixed crop-livestock rainfall sufficient zone. W: liveweight. Profit is from livestock production only. 1 USD = 22 ETB or 66 INR.

Region x scenario	Flock size (heads)	Births (heads)	Sales (heads)	Production (kg W/yr)	Mortality (%)	Annual profit
*Ethiopia - LG*						*ETB*
**Baseline**	**38.4**	**18.7**	**9.1**	**173**	**25**	**981**
Improved genetics	32.7	19.5	9.1	241	25	4,080
Low mortality	39.8	19.9	15.7	296	10	2,937
Improved genetics + improved pasture	39.5	24.5	11.4	316	25	6,756
Improved pasture	56.5	30.5	15.6	344	25	4,705
Improved genetics + low mortality	38.1	24.0	19.2	404	10	6,811
Improved genetics, improved pasture + low mortality	45.6	30.2	24.0	530	10	11,062
Improved pasture + low mortality	57.2	30.8	24.0	534	10	8,005
*Ethiopia – MRD*						*ETB*
Improved genetics	14.7	7.8	3.0	60	25	−562
**Baseline**	**18.3**	**9.0**	**4.6**	**69**	**23**	**−3**
Low mortality	20.7	11.4	8.7	108	12	257
Improved genetics + low mortality	16.7	10.0	5.9	110	17	264
Improved pasture + low mortality	22.0	14.2	11.6	201	10	1,239
Improved genetics, improved pasture + low mortality	17.9	11.3	8.3	207	11	2,095
*Ethiopia – MRS*						*ETB*
**Baseline**	**15.1**	**10.5**	**6.8**	**158**	**15**	**1,323**
Low mortality	15.9	11.2	8.9	201	8	2,307
Improved forage + low mortality	15.8	11.2	8.9	217	8	3,520
*India – Arid zone*						*INR*
**Baseline**	**10.2**	**4.9**	**2.9**	**33**	**25**	**3,251**
Baseline + low mortality	12.1	6.0	3.8	43	19	3,543
Free grazing	8.2	5.1	4.1	50	11	7,838
Supplement kids	10.2	4.9	2.9	57	25	5,496
Supplement does (cereal straw)	12.8	6.4	4.6	57	15	6,233
Improved pasture	13.0	6.9	5.0	62	15	8,375
Improved pasture + low mortality	13.5	7.4	6.1	71	9	8,803
Supplement does (wheat bran)	15.2	10.2	8.7	86	9	1,890

Farmers in the MRS zone had no access to pasture and it was assumed only crossbred goats would be kept. With baseline diets based on cereal and pulse straw and livestock fed adequately, a modest profit could be generated (Table 4). The profitability and productivity of goat production were both increased through improving healthcare, but the biggest improvements occurred when improved healthcare was coupled with better livestock nutrition. Feeding goats noug seed cake improved productivity by 37%, and although profitability was still modest, it was almost three times that of the baseline simulation.

## 4 India

Goat production was constrained by poor reproduction and high mortality rates, which led to low numbers of livestock available for sale or home consumption (Table 4). In addition, male offspring weighed only 9 kg when sold at 6 months.

Improving livestock nutrition increased production through higher reproduction and growth rates, and decreased mortality of both adults and kids. Supplementing does with wheat bran provided the largest increase in production. However, it was also the most expensive intervention, with a smaller profit than the baseline scenario. Providing wheat bran to does increased kidding and survival rates, but growth of weaned offspring, and therefore sale weights, remained low. Supplementing does with poorer quality cereal straw caused a smaller increase in livestock production, but was a more profitable feeding strategy. When supplement was directed towards weaned male goats, there was little change to flock mortality, but growth rates were much higher, and livestock were sold at an average of 21 kg. While improving livestock nutrition through unrestricted access to grazing land (lower stocking rates) or improved pastures resulted in smaller increases in production, these scenarios provided the biggest increases in household income.

Improved healthcare only caused a small decrease in mortality rates and minimal increase in profit when livestock nutrition was not also addressed (baseline + low mortality, Table 4). Improved health care had a much larger impact on production and income when combined with an intervention that also addressed goat nutrition (improved pasture + low mortality).

## 5 Discussion

Results from our analyses suggest that there is large potential to increase goat meat production by smallholder farmers in Ethiopia and India, with positive implications for household incomes. In extensive production systems (Ethiopia LG, MRD and India arid zone) goat meat production could be increased by up to 200% through combinations of improved nutrition, genetics and healthcare. Production increases were smaller in the intensive goat production system (Ethiopia MRS), but the baseline scenario included crossbred goats, so there was less scope to increase production.

A key finding to emerge from this study is that yield gaps will be best addressed by integrated technologies using a systems approach. It is highly unlikely that single “silver bullet” technologies have the ability to substantially lift productivity and profitability. As there are several key rate limiting steps to improved productivity i.e. nutrition, genetics and disease, these must be addressed concurrently. This is not a new finding, but is important to highlight. Unfortunately, many government and donor programs tend to focus on improving single components of the system and there is not the coordination to achieve the necessary integration that has the capacity to close yield gaps in ruminant livestock systems.

Another important outcome from our study is that interventions resulting in the biggest increases in goat meat production or number of animals sold do not always give the highest profits because input costs of some interventions can be high. This is significant because savings and cash income from livestock sales are often the primary reason for keeping goats (Hassen and Tesfaye, 2014, Tadesse et al., 2014). However, cash for inputs is often lacking so packages that increase household income with only modest additional inputs may be more attractive to smallholder farmers because cash can be spent on food, healthcare, education and other necessities. In addition, Ritzema et al. (2017) showed that it is sale of livestock products provide a greater contribution to household food security than home-consumption.

Our analysis shows that large increases in goat production and profitability are possible in extensive grazing systems, which is where most small ruminants are currently managed. However, increases will rely on improved management of communal grazing resources through reduced stocking rates and improved pasture condition. Decreasing grazing pressure on communal rangelands and careful livestock management are required to maintain rangeland condition and increase the amount of feed available per animal. This needs a sustained community level approach, and may require government incentives, combined with increased management and regulation of communal lands. While decreasing flock numbers can increase both production and profit at a household level, it may be a risky practice for smallholder farmers unless animal disease and mortality is also addressed.

Improving the quality of pasture by oversowing with a legume resulted in a higher profit compared to other interventions with similar levels of production because no cost to the farmer was included in the model. In practice, oversowing natural pastures on communal grazing land will require careful selection of appropriate species and developing reliable establishment techniques. This needs to be followed by careful management in the months following pasture introduction to ensure a high chance of successful establishment. On communal grazing lands, it will almost inevitably fall on government to improve pastures. The capital costs of pasture improvement on an area of land sufficient to sustain the modelled herd would be beyond the financial capacity of smallholder households based on returns determined in this study. Governments have been recommending oversowing native pastures/rangeland with introduced herbaceous legumes to improve pasture productivity and protein content of diets for many decades in east Africa, including the most recent Ethiopian Livestock Master Plan (Shapiro et al., 2015). While there has been technical success with oversowing legumes (e.g., Mengistu 2002), there is limited evidence of widespread adoption due to upfront establishment costs and risks associated with successful pasture establishment in a variable climate. There are also challenges with slow establishment of oversown legumes (Miller and Stockwell 1991) although there is evidence of successful establishment in African communal grazing lands (Annor and Cofie 2007).

Further increases in production were achievable in more intensive systems through supplementation with cereal straw and crop by-products (Table 4). Targeted supplementation of specific classes of livestock had a large impact, and would be most practical for farmers with stall-fed livestock. This is highlighted in the Indian example, where supplements could be provided to weaned male goats to increase growth and sale rates, or does to increase kidding and survival rates. If resources were available, an effective strategy might be to feed poor quality crop residues to mature does, which have relatively low energy requirements, whilst directing higher quality but more expensive supplements towards male goats, which can be sold for cash (e.g., Mayberry et al., 2016).

Whilst the opportunities to lift ruminant productivity through improved forages and/or feeding appear compelling from the analysis in this study, the challenges associated with adoption and implementation should not be under-estimated. Owen et al. (2012) in reviewing limited success of animal nutrition interventions in developing countries identified several causes including: poor or inappropriately targeted extension efforts; lack of participatory research and development approaches; and inadequate demonstration of benefit: cost ratios.

Even with improved animal nutrition, the low genetic potential of local goat breeds mean that large increases in production at the farm scale are not possible without improved genetics. Improving livestock genetics is a popular strategy with donors, and in the right circumstances can lead to substantial increases in production and profit. Leroy et al. (2016) provide a review of some of the challenges affecting the success of genetic improvement programs in developing countries. These include appropriate animal management and nutrition, adaptation of exotic breeds to challenging environmental conditions and diseases, the logistics of developing and maintaining systems for distributing improved genetics (e.g., artificial insemination), and costs associated with investing in new genetics (animals and infrastructure).

Reducing animal mortality rates through better healthcare and disease management provided a relatively low risk option to increase production rates and household income (Table 4). Lower mortality rates resulted in larger flock sizes, thus a higher number of births per year and more animals available for sale. The biggest increases in production and profit were achieved when low mortality rates were combined with improved nutrition and better genetics, so that increased animal numbers were accompanied by increased sale weights. It is also worth considering that increasing the flock size through reduced disease and mortality rates will increase the resource requirements (feed and labour) of smallholder famers.

Whilst mortality rates in goats are high (Gizaw et al., 2010), a limitation of this analysis was the paucity of data to confidently parameterise the model for the mortality impacts of specific diseases. Overall mortality rates from disease and management complexes were instead used. Further, there is little information available on how diseases affect growth and production in those animals that remain alive. The productivity improvements beyond mortality reduction were therefore not considered in the reduced mortality scenarios, which may underestimate the benefits of disease reduction. More effort needs to be directed to better quantifying the benefits of disease management.

## 6 Conclusion

While government services and development programs are often biased against goats in favour of large ruminants, our results show that there is value to smallholders in investments in small ruminants. Household modelling showed that reproduction, growth and survival rates can be increased through better nutrition, genetics and healthcare, but that the biggest increase in production and profits will occur when multiple interventions are combined.
